# New combined microRNA and protein plasmatic biomarker panel for pancreatic cancer

**DOI:** 10.18632/oncotarget.12406

**Published:** 2016-10-03

**Authors:** Wei Yuan, Wanyan Tang, Yibin Xie, Shaoming Wang, Yingtai Chen, Jun Qi, Youlin Qiao, Jie Ma

**Affiliations:** ^1^ State Key Laboratory of Molecular Oncology, National Cancer Center/Cancer Hospital, Chinese Academy of Medical Sciences, Peking Union Medical College, Beijing 100021, China; ^2^ Department of Abdominal Surgical Oncology, National Cancer Center/Cancer Hospital, Chinese Academy of Medical Sciences, Peking Union Medical College, Beijing 100021, China; ^3^ Department of Epidemiology, National Cancer Center/Cancer Hospital, Chinese Academy of Medical Sciences, Peking Union Medical College, Beijing 100021, China; ^4^ Department of Clinical Laboratory, National Cancer Center/Cancer Hospital, Chinese Academy of Medical Sciences, Peking Union Medical College, Beijing 100021, China; ^5^ Clinical Immunology Center, Chinese Academy of Medical Science, Beijing, 100730, China

**Keywords:** pancreatic cancer, plasma miRNAs, MIC-1, diagnosis, sensitivity and specificity

## Abstract

**Introduction:**

Lack of diagnostic makers results in loss of operation opportunity in that most patients are diagnosed at the late stage. Pancreatic cancer (PC) has been regarded as a fatal disease with a 5-year survival rate below 10%. Therefore, the development of diagnostic biomarkers for PC is in urgent need to control the mortality of the disease.

**Materials and Methods:**

This is a case-control study including 640 plasma samples from healthy controls (HC), patients with benign pancreatic diseases (BPD), patients with PC; and patients with other gastrointestinal (GI) cancers. Eight biomarker candidates, including miR-20a, miR-21, miR-25, miR-155, miR-196a, miR-210, Macrophage Inhibitory Cytokine-1(MIC-1) and CA19-9, were evaluated to establish two diagnostic indexes in this study.

**Results:**

The plasma level of the six miRNAs and MIC-1, CA19-9 were elevated in PC patients compared with those of healthy controls (P<0.001). Among them, miR-20a, miR-21, miR-25, MIC-1 and CA19-9 could distinguish PC patients from those with other GI cancers or BPD. With multivariable logistic regression, we established two specific indexes for diagnosis of PC(Index1 contains miR-21, MIC-1 and CA19-9; Index2 contains miR-25, MIC-1 and CA19-9). In a randomized setting of 260 HC, 168 PC, 132 other GI cancers and 80 BPD patients, both indexes performed not only better sensitivity for PC but also better specificity to distinguish PC from other GI cancers than CA19-9 and individual biomarkers.

**Conclusions:**

These results indicated that combination of biomarkers as a panel could improve diagnostic values compared with using a single marker. Such panels as illustrated in this study could provide novel plasmatic biomarker for PC diagnosis.

## INTRODUCTION

Pancreatic cancer (PC) is a highly malignant cancer with a 5-year survival rate below 10% because of lack of symptoms at its early stage and effective systemic therapies [1]. Surgery is considered as the only curative intervention, which can only be used at early stage of the disease. Because of the shortage of screening methods for early detection, the mortality of this disease has not changed over the past few decades [2]. Therefore, discovery of blood biomarkers to identify pancreatic cancer patients at an early stage will be the key to control the mortality of pancreatic cancer.

Currently, CA19-9 is the only serum detectable protein used to monitor the progress of PC in clinic [3]. Since cancer development is a complicated process with alterations of numerous cancer-related genes and pathways, a single biomarker like CA19-9 could hardly provide complete information about the disease development [4]. It is likely that combination of multiple biomarkers could provide the most accurate tool for PC diagnosis.

Macrophage Inhibitory Cytokine-1 (MIC-1/GDF15), a secretary form of the transforming growth factor-β (TGF-β) superfamily, was reported to increase in tissues and serum/plasma of PC patients [5–8]. Although MIC-1/GDF15 seems to be a very promising diagnostic candidate for PC, there is limited data available on the performance of MIC-1 in cohort study.

MicroRNAs were reported to play an important role in cancer development. Altered expression of microRNAs in human serum or plasma was identified in different types of cancer [9–13]. With a high stability in body fluids, microRNAs are expected to be promising biomarkers for cancer diagnosis. In the past few years, miR-20a, miR-21, miR-25, miR-155, miR-196a, and miR-210 were reported to be overexpressed in pancreatic cancer tissue and elevated in patient serum or plasma [14–18]. Most of these studies were conducted in Caucasians (vs. Asians). However, diagnostic value and specificity of these microRNAs for PC in Asians are unknown.

In the present study we evaluated the diagnostic values (sensitivity and specificity) of six plasma miRNAs (including miR-20a, miR-21, miR-25, miR-155, miR-196a, and miR-210), as well as MIC-1 and CA19-9 for pancreatic cancer. The six microRNAs were selected as biomarker candidates according to the following criterion: highly expressed in PC tissues and detectable in the plasma/serum in PC patients as previously reported. We established two combined indexes to test our hypothesis that a panel of biomarkers has better performance than a single biomarker in cancer diagnosis. We collected plasma samples from patients with benign pancreatic disease, other gastrointestinal cancer in order to develop a diagnostic index with disease specificity. A blinded validation group was used to evaluate the diagnostic values of the established indexes in case of biased conclusions. An independent predictive double-blinded test was further conducted to detect the accessibility of the indexes to PC screening as well.

## RESULTS

There were 640 samples prepared and used in this study. Summary of characteristics of study participants is shown in Table 1 and supplementary Table 1. In the case-control study, age, gender, cigarette smoking, alcohol drinking, hypertension, diabetes, body mass index (BMI) or cancer heritage did not show a significant association comparing the PC patients and the healthy control subjects, though smoking status and diabetes were considered to be risk factors for pancreatic cancer [2]. Among younger patients with chronic pancreatitis and benign pancreatic tumor, there was a significant difference in age (*P*=0.002 in the training group and *P*<0.001 in the validation group) between PC patients and those with benign pancreatic disease. Since most of the GI cancer samples were obtained from tumor resection, rate of tumor resection and cancer stage were found to be associated with PC compared with patients with other gastrointestinal cancers both in the training (*P*=0.003) and blinded validation group (*P<*0.001).

**Table 1:** Comparison of basic characteristics between PC patients and other groups in the training group and blinded validation group

	No. (%) of Patients and Healthy Participants of each group				*P* value			
	PC(n=164)	HC(n=260)	BPD(n=80)	Other GI cancer(n=132)	PC VS HC	PC VS BPD	PC VS HC+BPD	PC VS Other GI cancer
**Training group (n = 240)**	**76(31.67)**	**82(34.17)**	**22(9.17)**	**60(24.99)**				
Age, median (range),y	61.00(32.00 – 82.00)	58.00(43.00-81.00)	52.00(21.00-80.00)	61.00(34.00-76.00)	0.538^a^	0.002^a^	0.061^a^	0.601^a^
Gender					0.454^b^	0.137^b^	0.262^b^	0.208^b^
Male	48(63.16)	47(57.32)	10(45.45)	44(73.33)				
Female	28(36.84)	35(42.86)	12(54.55)	16(26.67)				
Resection of tumors								<0.001^b^
Yes	12(15.79)	/	/	57(95.00)				
No	64(84.21)	/	/	3(5.00)				
Metastasis								0.125^b^
Yes	33(43.42)	/	/	34(56.67)				
No	43(56.58)	/	/	26(43.33)				
Cancer stage								0.003^c^
I	4(5.26)	/	/	13(21.67)				
II	8(10.53)	/	/	10(16.67)				
III	46(60.53)	/	/	29(48.33)				
IV	18(23.68)	/	/	8(13.33)				
Hypertension					0.314^b^	0.618^b^	0.311^b^	0.316^b^
Yes	25(32.89)	21(25.61)	6(27.27)	15(25.00)				
No	51(67.11)	61(74.39)	16(72.73)	45(75.00)				
Diabetes					0.642^b^	0.974^b^	0.691^b^	0.204^b^
Yes	21(27.63)	20(24.39)	6(27.27)	11(18.33)				
No	55(72.37)	62(75.61)	16(72.73)	49(81.67)				
Cancer heritage					0.518^b^	0.058^b^	0.992^b^	0.725^b^
Yes	11(14.47)	15(18.29)	0(0.00)	10(16.67)				
No	65(85.53)	67(81.71)	22(100.00)	50((83.33)				
Smoking					0.129^b^	0.629^b^	0.152^b^	0.656^b^
Yes	32(42.11)	25(30.49)	8(36.36)	23(38.33)				
No	44(57.89)	57(69.51)	14(63.64)	37(61.67)				
Alcohol drinking					0.124^b^	0.308^b^	0.100^b^	0.245^b^
Yes	26(34.21)	19(35.37)	5(22.73)	15(25.00)				
No	50(65.79)	63(64.63)	17(77.27)	45(75.00)				
BMI, median (range)	24.11(16.98-35.41)	24.31(18.13-33.65)	23.66(19.49-28.73)	24.69(19.36-31.20)	0.505^a^	0.553^a^	0.724^a^	0.608^a^
Plasma CA19-9, median(range), KU/L	365.70(0.927-28840.0)	11.21(0.60-32.21)	22.03(2.96-53.9)	15.71(4.51-315.00)	/	/	/	/
Serum CEA, median(range), μg/L	4.80(0.6-271.40)	2.00(0.258-4.95)	1.00(1.00-4.86)	2.45(0.22-251.40)				
**Validation group (n = 280)**	**82(29.28)**	**88(31.43)**	**50(17.86)**	**60(21.43)**				
Age, median (range)	59.00(35.00-88.00)	59.00(43.00-82.00)	49.00(16.00-71.00)	56.00(30.00-83.00)	0.493^a^	<0.001^a^	0.046^a^	0.575^a^
Gender					0.270^b^	0.206^b^	0.173 ^b^	0.938^b^
Male	47(57.32)	43(48.86)	23(46.00)	34(56.67)				
Female	35(42.68)	45(51.14)	27(54.00)	26(43.33)				
Resection of tumors								<0.001^b^
Yes	8(9.76	/	/	60(100.00)				
No	74(90.24)	/	/	0(0.00)				
Metastasis								0.904^b^
Yes	35(42.68)	/	/	25(41.67)				
No	47(57.32)	/	/	35(58.33)				
Cancer stage								<0.001^c^
I	1(1.22)	/	/	14(23.33)				
II	7(8.54)	/	/	21(35.00)				
III	51(62.20)	/	/	22(36.67)				
IV	23(28.05)	/	/	3(5.00)				
Hypertension					0.186^b^	0.581^b^	0.232^b^	0.620^b^
Yes	25(30.49)	19(21.59)	13(26.00)	16(26.67)				
No	57(69.51)	69(78.41)	37(74.00)	44(73.33)				
Diabetes					0.241^b^	0.060^b^	0.080^b^	0.203^b^
Yes	21(25.61)	16(18.18)	6(12.00)	10(16.67)				
No	61(74.39)	72(81.82)	44(88.00)	50((83.33)				
Cancer heritage					0.683^b^	0.191^b^	0.367^b^	0.114^b^
Yes	13(15.85)	12(13.64)	4(8.00)	16(26.67)				
No	69(84.15)	76(86.36)	46(92.00)	44(73.33)				
Smoking					0.139^b^	0.331^b^	0.507^b^	0.408^b^
Yes	17(20.73)	27(30.68)	7(14.00)	16(26.67)				
No	65(79.27)	61(69.32)	43(86.00)	44(73.33)				
Alcohol drinking					0.374^b^	0.197^b^	0.920^b^	0.233^b^
Yes	15(18.29)	21(23.86)	5(10.00)	16(26.67)				
No	67(81.71)	67(76.14)	45(90.00)	44(73.33)				
BMI, median (range)	24.19(16.14-30.15)	23.77(18.59-36.65)	24.58(16.46-30.83)	24.22(16.82-32.01)	0.433^a^	0.120^a^	0.196^a^	0.665^a^
Plasma CA19-9, median(range), KU/L	103.41(0.60-32127.00)	10.29(0.60-41.23)	15.86(0.60-20.12.00)	15.85(1.12-1038.40)	/	/	/	/
Serum CEA, median(range) μg/L	3.93(0.84-681.30)	2.01(0.77-6.21)	1.40(0.36-17.38)	2.01(0.56-38.85)	/	/	/	/

a Student's T-test.

b chi-square.

c Mann-Whitney U.

Abbreviation: PC, pancreatic cancer; HC, healthy controls; BPD, benign pancreatic disease (including chronic pancreatitis and benign pancreatic tumor); GI, gastrointestine.

**Figure 1:**
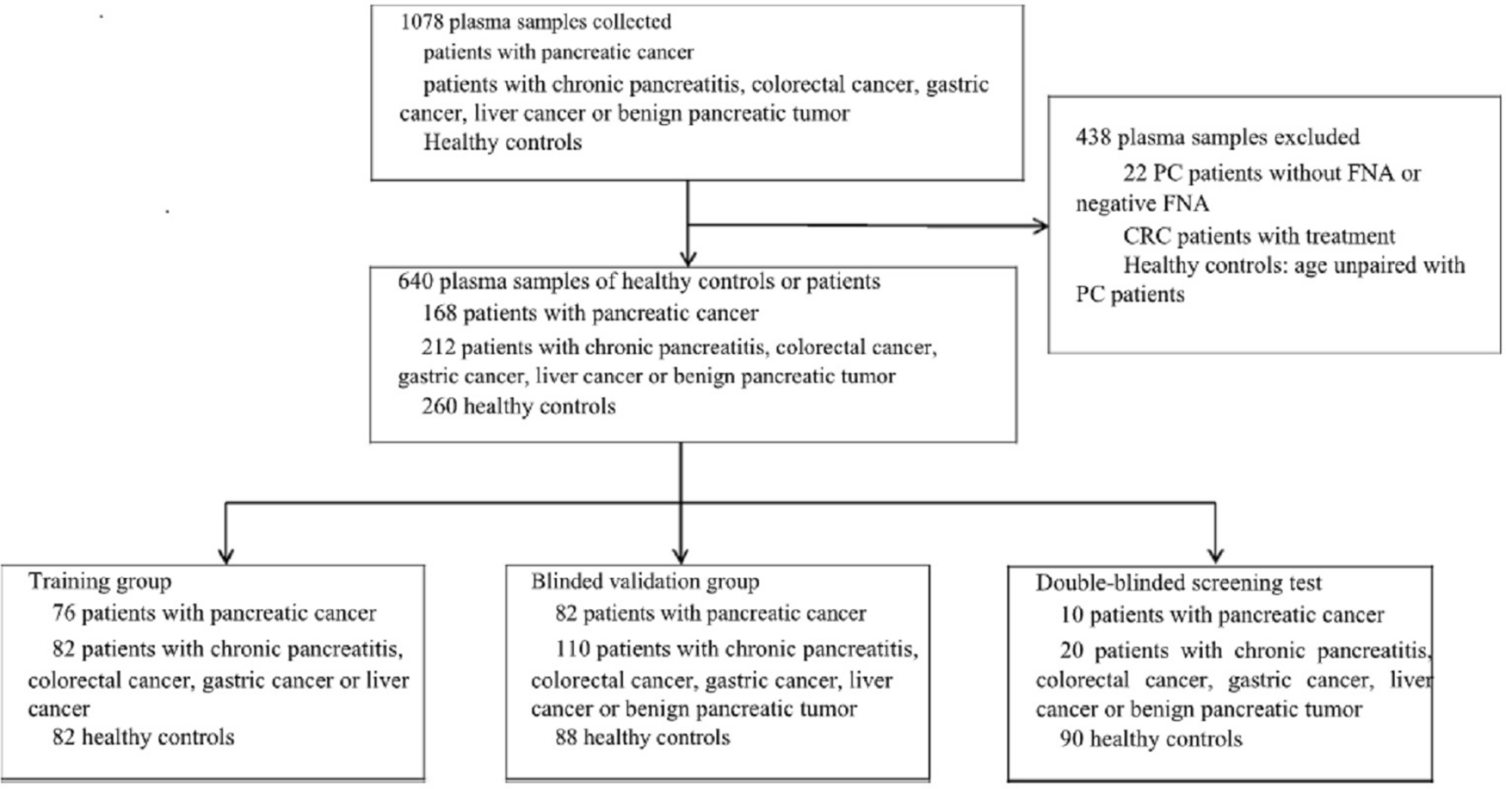
Box plots with traditional Tukey whiskers, showing 1. 5 times the interquartile distance The horizontal line in the middle of each box indicates the median, and the points beyond the whiskers are outliers. All data shown in this figure was Log(e)-transformed from the copy numbers of miRNAs in each microliter of plasma samples and the expression concentration of MIC-1 (pg/ml) and CA19-9(KU/L). The expression levels of MIC-1, miR-20a, miR-21 and miR-25 were significantly up-regulated in PC patients when compared with healthy controls (all, *P*<0.001) and those with either CP or other GI cancers. There's no significant difference in the expression of miR-155 and miR-210 between PC patients and other patients. The expression of miR-196a was lower in the PC patients than that in other GI cancer patients (*P*=0.030).* *P*<0.05, **P<0.01, ***P<0.001.

In the training group, levels of miR-20a, miR-21, miR-25, miR-155, miR-196a, miR-210, MIC-1 and CA19-9 were significantly higher in the plasma of patients with pancreatic cancer compared with those of healthy controls (Figure 1, *P*< 0.001). Among them, miR-20a, miR-21, miR-25, MIC-1 and CA19-9 were specifically elevated in PC patients compared with CP patients.

We further detected all seven candidate biomarkers specifically expressed in the plasma of other GI cancer patients (Figure 1). It was found that the expression of miR-20a, miR-21, miR-25, MIC-1 and CA19-9 was elevated in the plasma of PC patients compared with other GI cancers. However, miR-196a was down-regulated. There was no significant difference in the expression of miR-155 and miR-210 between PC patients and other GI cancer patients.

The ability of each tissue specific biomarker to distinguish PC patients from healthy controls and other patients was assessed by using Binary Logistic regression (Supplementary Table 2). Univariate logistic regression analysis showed that miR-20a, miR-21, miR-25, MIC-1 and CA19-9 had the potential to differentiate PC patients from healthy controls or CP patients (all, OR>1, *P*< 0.001) in the training group. On the contrary, miR-196a could not differentiate pancreatic cancer from chronic pancreatitis (*P*=0.536). Although miR-196a could distinguish PC from other GI cancers (*P*=0.030), the odds ratio was less than 1 (0.728, 95%CI: 0.547-0.969). Since miR-20a, miR-21, miR-25, MIC-1 and CA19-9 could distinguish PC patients from other diseases, they were further calculated to develop specific combined indexes for PC diagnosis using multivariable regression analysis (Supplementary Table 3).

Based on the results from the training group (PC patients VS Healthy controls & CP patients), two combined diagnostic indexes were developed. Index 1 was (1.795 x miR-21) + (1.971 x MIC-1) + (1.020 x CA19-9) - 42.305. Index 2 was (1.772 x miR-25) + (2.138 x MIC-1) + (1.125 x CA19-9) - 45.006.

As presented in Table 2, the AUC of either Index 1 (*P*=0.001) or Index 2 (*P*=0.001) was larger than CA19-9 when detecting PC patients against healthy controls and other patients. In the training group, the AUC was 0.968 (95%CI, 0.947-0.989) for Index 1, 0.967 (95%CI, 0.945-0.989) for Index 2, and 0.895 (95%CI, 0.838-0.952) for CA19-9. The sensitivity of Index1, Index2 and CA19-9 were 0.895, 0.895 and 0.816 respectively; the specificity were 0.909, 0.915 and 0.933 respectively. The accuracy was 0.904 for Index1, 0.908 for Index2 and 0.892 for CA19-9. Both indexes had better performance in AUC, sensitivity, accuracy and negative predictive value (NPV) than each single biomarker in the training group (Data not shown) though only AUC had significant difference. When testing pancreatic cancer patients against those with benign pancreatic diseases, both Index1 and Index2 performed better AUC, sensitivity, specificity, accuracy and NPV than CA19-9 alone. Thus, the panel of the biomarkers significantly improved the diagnostic sensitivity and accuracy.

**Table 2:** The diagnostic value of combined Index in Training and Validation group

	Group	Factor	AUC-ROC(95%CI)	Sensitivity	Specificity	Accuracy	PPV	NPV	+LR	-LR
PC VS non-PC	Training Group	CA19-9	0.895(0.838-0.952)	0.816	0.933	0.892	0.838	0.916	12.179	0.197
		Index1(miR-21,MIC-1,CA19-9)	0.968(0.947-0.989)**	0.895	0.909	0.904	0.810	0.949	9.835	0.116
		Index2(miR-25,MIC-1,CA19-9)	0.967(0.945-0.989)**	0.895	0.915	0.908	0.829	0.949	10.529	0.115
	Blinded validation Group	CA19-9	0.862(0.809-0.915)	0.720	0.859	0.818	0.678	0.881	5.106	0.326
		Index1(miR-21,MIC-1,CA199)	0.915(0.878-0.953)*	0.878*	0.874	0.875	0.742	0.945	6.968	0.140
		Index2(miR-25,MIC-1,CA199)	0.920(0.883-0.957)*	0.841	0.919	0.896*	0.812	0.933	10.383	0.173
PC VS HC	Training Group	CA19-9	0.914(0.861-0.968)	0.816	1.000	0.911	1.000	0.854	+	0.184
		Index1(miR-21,MIC-1,CA19-9)	0.990(0.981-1.000)**	0.895	0.988	0.943	0.986	0.910	74.583	0.106
		Index2(miR-25,MIC-1,CA19-9)	0.984(0.970-0.998)**	0.895	0.988	0.943	0.986	0.910	74.583	0.106
	Blinded validation Group	CA19-9	0.908(0.860-0.957)	0.720	0.955	0.841	0.937	0.785	16.000	0.293
		Index1(miR-21,MIC-1,CA19-9)	0.972(0.946-0.999)**	0.878*	0.989	0.935*	0.986	0.897	79.818	0.123
		Index2(miR-25,MIC-1,CA19-9)	0.967(0.938-0.995)**	0.841	1.000*	0.924*	1.000	0.871	+□	0.159
PC VS BPD ^a^	Training Group	CA19-9	0.874(0.806-0.941)	0.816	0.773	0.806	0.925	0.548	3.595	0.238
		Index1(miR-21,MIC-1,CA19-9)	0.938(0.893-0.983)*	0.895	0.818	0.878	0.944	0.692	4.918	0.128
		Index2(miR-25,MIC-1,CA19-9)	0.939(0.894-0.984)*	0.895	0.818	0.878	0.944	0.692	4.918	0.128
	Blinded validation Group	CA19-9	0.821(0.749-0.894)	0.720	0.800	0.750	0.855	0.635	3.600	0.350
		Index1(miR-21,MIC-1,CA19-9)	0.889(0.831-0.947)*	0.878*	0.860	0.871*	0.911	0.811	6.271	0.142
		Index2(miR-25,MIC-1,CA19-9)	0.865(0.799-0.930)	0.841	0.860	0.848*	0.908	0.768	6.007	0.185
PC VS other gastrointestinal cancer	Training Group	CA19-9	0.876(0.813-0.939)	0.816	0.883	0.846	0.899	0.791	6.974	0.208
		Index1(miR-21,MIC-1,CA19-9)	0.948(0.914-0.981)*	0.895	0.817	0.860	0.861	0.860	4.891	0.129
		Index2(miR-25,MIC-1,CA19-9)	0.954(0.923-0.985)**	0.895	0.850	0.875	0.883	0.864	5.967	0.124
	Blinded validation Group	CA19-9	0.829(0.761-0.897)	0.720	0.767	0.739	0.808	0.667	3.090	0.365
		Index1(miR-21,MIC-1,CA19-9)	0.853(0.791-0.915)	0.878*	0.717	0.810	0.809	0.811	3.102	0.170
		Index2(miR-25,MIC-1,CA19-9)	0.886(0.831-0.940)	0.841	0.850	0.845*	0.885	0.797	5.607	0.187

a BPD: Benign pancreatic disease. In the Training group only chronic pancreatitis patients were included. In the Blinded validation group patientswith both chronic pancreatitis and benign pancreatic tumor were included.

* *P* <0.05 when compared with CA19-9;

** *P*<0.005 when compared.

Non-PC group included healthy controls, chronic pancreatitis, benign pancreatic tumor, colorectal cancer, gastric cancer and liver cancer; Abbreviation:PPV, Positive predictive value; NPV, Negative predictive value;+LR, Positive Likelihood Ratio; -LR, Negative Likelihood Ratio.

**Figure 2:**
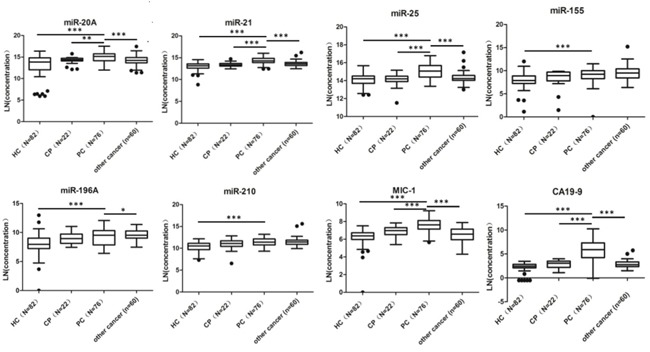
Receiver operating characteristics (ROC) curve analysis (A) and box plots (B) of Index 1, 2 in Training Group and Validation Group for discriminating PC from non-PC **A.** Index1, Index2 and CA19-9 yielded AUCs of 0.968 (95%CI, 0.947-0.989), 0.967 (95%CI, 0.945-0.989) and 0.895 (95%CI, 0.838-0.952) respectively, in the training group. In the validation group, the AUC was 0.915 (95%CI, 0.878-0.953) for index1, 0.920 (95%CI, 0.883-0.957) for index2 and 0.862 (95%CI, 0.809-0.915) for CA19-9 respectively. **B.** Box plots with traditional Tukey whiskers, showing 1.5 times the interquartile distance. The horizontal line in the middle of each box indicates the median, and the points beyond the whiskers are outliers. The box plots of both indexes showed significant difference between PC group and non-PC group (***, *P*<0.001).

In order to avoid biased conclusions from the training group, expression of miR-21, miR-25 and MIC-1, CA19-9 was determined in a blinded validation group. Samples from patients with benign pancreatic tumors were also included in the blinded validation group. The values of the univariate logistic regression analysis for each biomarker in the validation group are shown in Supplementary Table 2. In the blinded validation group (Table 2), using the indexes to diagnose PC patients from all non-PC controls, the AUC was 0.915 (95%CI, 0.878-0.953) for Index 1(*P*=0.029), 0.920 (95%CI, 0.883-0.957) for Index 2(*P*=0.014), and 0.862 (95%CI, 0.809-0.915) for CA19-9. The sensitivity was 0.878 for index1 (*P*=0.016), 0.841 for index2 (*P*=0.059) and 0.720 for CA19-9. The specificities were 0.874, 0.919 (*P*=0.055) and 0.859, respectively. The accuracy was 0.875 (*P*=0.061), 0.896 (*P*=0.008), 0.818, respectively. In the validation group, both indexes performed better in terms of AUC, sensitivity, specificity and accuracy than each single biomarker when diagnosing PC from non-PC, healthy controls or benign pancreatic diseases. The ROC curves and box plots of Index 1 and Index 2 in the PC group and non-PC group were shown in Figure 2.

To validate the utility of our selected indexes for PC diagnosis and screening, we performed a double-blinded screening test in PC patients, healthy controls and patients with other diseases. Based on the molecular expression in plasma, 9 of 10 PC patients were diagnosed either with the indexes or CA19-9. As shown in Table 3, Index 1 (0.955, *P*=0.077) and Index 2 (0.964, *P*=0.038) performed better specificity than CA19-9 (0.891). In addition, both indexes showed better diagnostic specificity and accuracy than CA19-9 alone.

**Table 3:** Diagnostic sensitivity and specificity in the Double-blinded test

	Positive/True positive	Sensitivity	Negative/True Negative	Specificity	Accuracy	FPR	FNR	PPV	NPV
Index1	9/10	0.900	105/110	0.955	0.950	0.045	0.100	0.643	0.991
Index2	9/10	0.900	106/110	0.964	0.958	0.042	0.100	0.600	0.991
CA19-9	9/10	0.900	98/110	0.891	0.892	0.109	0.100	0.428	0.990

Abbreviation: FPR, False positive rate; FNR, False negative rate; PPV, Positive predictive value; NPV, Negative predictive value.

As most pancreatic patients were diagnosed at advanced stage, all patients with low-stage pancreatic cancer from the three groups were pooled (stage I and II, n=21) to assess the performance of Indexes. The sensitivity of Index1, Index2 and CA19-9 were reduced to 0.762, 0.810 and 0.714, respectively (Data not shown). However, new indexes had better performance than CA19-9 as early diagnostic tools for pancreatic cancer. As illustrated in supplementary Table 4, two new indexes could diagnose not only PC patients with positive CA19-9, but also those with negative CA19-9. In the training group, both indexes identified 8 out of 14 CA19-9 negative PC patients. In the blinded validation group, 17 and 16 out of 23 were identified by Index I and II, respectively.

A relationship between the expression of candidate biomarkers and clinical characteristics of PC was analyzed in 168 patients. We found that the expression levels of miR-20a, miR-21, miR-25, miR-210, MIC-1 and CA19-9 had no significant correlation with the clinical characteristics of PC patients (Supplementary Table 5). The expression levels of miR-155 were higher in PC patients at advanced stage than those at low-stage whose tumors were resectable. There is significant difference in miR-196a level between patients with or without distant metastasis. It is notable that Index2 performed significant difference between patients with or without hypertension (*P*=0.027) and diabetes (*P*=0.030), and correlation with the age of PC patients (ρ=0.172, *P*=0.025).

Kaplan-Meier survival analysis was conducted to investigate the prognostic value of the seven candidate biomarkers and the two combined indexes. Of the total of 113 PC patients in the training and validation group, 16 patients failed to follow-up. Analysis of 97 PC patients found that all biomarkers and indexes could not predict the survival rate of the patients (Supplementary Table 6).

## DISCUSSION

The purpose of this study was to explore plasma biomarker panels for identification of PC patients as a first-line examination. All candidate microRNAs were reported highly expressed in pancreatic cancer tissues [14, 16, 17, 19] and in plasma/serum of PC patients [15, 17, 18, 20] in different investigations. Most of the studies were conducted in Caucasians but few in Asians. Among them, only miR-20a has been reported to be under-expressed in FNA samples of PC compared with benign tissues [21]. MiR-155 [17] and miR-196a [24] were found to be up-regulated in the precursor lesions of PC such as PanIN or IPMN. In addition to CA19-9, a conventional protein used to monitor the effect of treatment on PC patients, MIC-1 was included in order to achieve novel combination effect.

Among six selected microRNAs, some were also elevated in other types of cancer. For example, circulating miR-21 has been well studied in various cancers such as lung, liver, prostate, pancreatic cancer and glioma [22]. In order to obtain a panel which could distinguish PC from other cancers, tissue specificity became another important issue for us to select a biomarker for setting up the panel. Therefore, patients with other GI cancers were recruited into our study. In the training group, all microRNAs and MIC-1 were elevated in PC patients compared with healthy controls. This was consistent with previous reports [5–8, 14, 15]. However, when comparing the expression level in patients with PC, CP and other GI cancers, miR-210, miR-155 and miR-196a were pointed out to lose differentiate ability because of its overexpression in all [19, 21]. Eventually, miR-21, miR-25, miR-20a and MIC-1 were selected to build a diagnostic index. Two novel diagnostic indexes were established, in which miR-20a was ruled out in that it made no difference in the two indexes.

CA19-9 is the only serum biomarker approved by the FDA for pancreatic cancer. In clinic, CA19-9 is usually used to monitor chemoresponse and predict recurrence of PC. In this study, plasma CA19-9 was detected, which proved to have no difference in sensitivity and specificity for PC diagnosis compared with serum CA19-9 (Supplementary Figure 1).

Both indexes performed better sensitivity, specificity and accuracy than each single biomarker in the training group, validation group and double-blinded test. The results indicated that combination of biomarkers as a panel could improve diagnostic values compared with using a single marker.

In the double blinded test, one PC patient was not diagnosed because of low expression of microRNAs and proteins in the plasma. This phenomenon was also observed in Schultz's study [23]. In their discovery cohort, 2 PC patients were listed as Outliers who had undetectable microRNAs. If we had excluded this patient from detectable category, it would have increased the sensitivity of our indexes to 1.000. Nevertheless, a lower false positive rate and a higher positive predictive value support the use of these indexes in risk assessment for patients with pancreatic cancer (Table 3).

Although several studies found a prognostic value for miR-155, miR-196a, miR-210 and miR-21 [8, 7, 20, 25], we demonstrated that the candidate biomarkers had no association with disease development. A larger population and longer observation period are needed to investigate their prognostic potential.

In an attempt to find PC biomarkers, Wang et al. investigated circulating microRNAs in pancreatic juice and identified miR-205, miR-210, miR-492 and miR-1247 in pancreatic juice as promising diagnostic and prognostic biomarkers of pancreatic cancer [25]. Biomarkers in pancreatic juice exhibited good sensitivity and specificity, but the samples are hard to collect, especially form healthy individuals. In addition to pancreatic juice, several studies analyzed biomarkers in whole blood or serum-exosomes [23, 26]. Whole blood derived microRNAs contain the information related to patient's reaction to cancer, which might complicate the diagnostic decision. Exploration of biomarkers in serum-exosomes has become a hot issue recently, but its application in clinic is costly. Here, we identified panels of biomarkers in the plasma. Two biomarker panels had been established which might be candidates as PC diagnostic tools for the future clinical use.

Results of our study were limited by sample size and insufficient samples from early stage PC, because of the low incidence of pancreatic cancer, similar disadvantages existed in Bloomston and Marion's investigations [14, 15]. The significance of our indexes for early diagnosis is yet to be identified. Further investigations are required to include more samples and evaluate the indexes before clinical application.

In conclusion, we identified two biomarker combined panels in plasma of PC patients, which had a better performance than each single component. The panels had a high specificity to pancreatic tissue compared with other GI cancers. In blinded validation and application studies, both indexes showed better sensitivity and specificity than CA19-9. These novel indexes may provide a promising PC diagnostic tool which is worth further validation.

## MATERIALS AND METHODS

More detailed methods are provided in the Supplemental Experimental Procedures.

### Patient population

This study was performed according to the Reporting Recommendations for Tumor Marker Prognostic Studies (REMARK) guidelines [27]. A total of 1078 plasma samples were collected at two medical centers, Cancer Hospital of Chinese Academy of Medical Sciences (CHCAMS) and Peking Chaoyang Hospital, from March 2012 to May 2015. All blood samples were collected from the patients who were newly diagnosed and treatment naïve, with the approval from the Institutional Review Board (Approval NO: NCC2015SZ-03). Patients with acute infectious biliary or pancreatic disease were excluded. Lipemic and hemolyzed blood samples were rejected (Hemolysis was assessed based on visual inspection.). In this study benign pancreatic tumors consisted of serous cystadenoma of pancreas, pancreatic neuroendocrine tumors and solid pseudopapillary tumor of pancreas. Eventually, 380 patients (including 168 patients with pancreatic cancer, 32 patients with benign pancreatic tumor, 44 patients with colorectal cancer, 44 patients with gastric cancer, 44 patients with liver cancer and 48 patients with chronic pancreatitis) and 260 disease-free healthy donors were recruited in this study.

The final diagnosis of PC was based on the histological evaluation of surgically resected tissue specimens, cytological evaluation of intraoperative fine needle biopsy (FNA) or endoscopic ultrasound guided fine needle biopsy (EUS-FNA).

One hundred and thirteen pancreatic cancer patients were followed up after collection of their blood samples, the follow-up lasted at least 11.2 months until patients died. Ninety-seven patients were included in the final survival analysis while 16 patients were lost to follow-up.

### Study design

**Figure 3:**
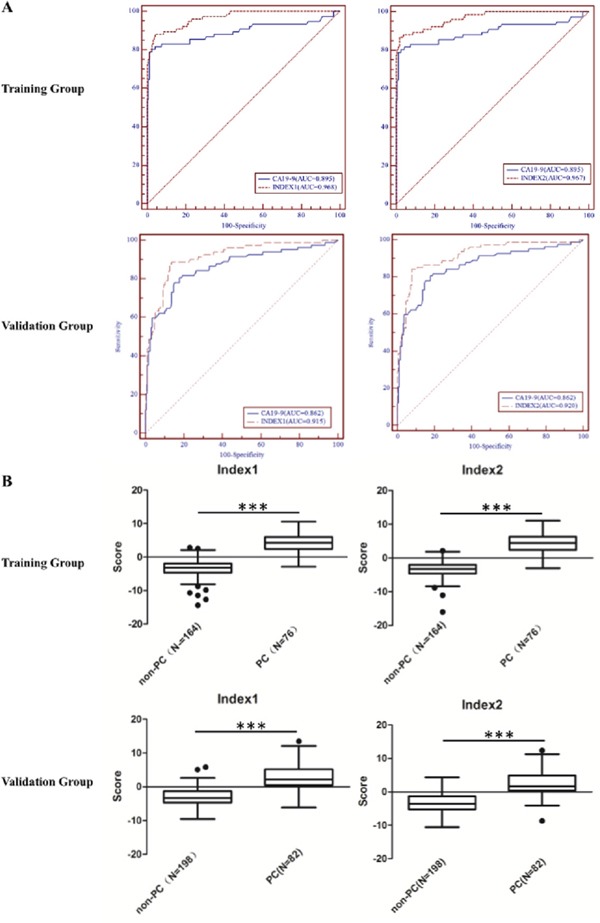
All groups consisted patients with pancreatic cancer, chronic pancreatitis, benign pancreatic tumor (BPT), colorectal cancer, gastric cancer, liver cancer and healthy controls BPT was not included in the Training group.

The study design is presented in Figure 3. This study consisted of a training group, a blinded validation group, as well as an independent double-blinded test group. Since circulating microRNAs and proteins in the blood can originate from tumor tissue, we selected six microRNAs and two proteins as biomarker candidates according to the following criterion: highly expressed in PC tissues and detectable in the plasma/serum in PC patients as previously reported.

The training group consisted of 76 patients with pancreatic cancer, 22 patients with chronic pancreatitis, 82 healthy control subjects and 20 patients with colorectal cancer, gastric cancer or liver cancer each. The selection of valuable biomarkers (among six miRNAs, MIC-1 and CA19-9) to establish multivariable logistic regression models was performed in the training group.

The blinded validation group consisted of 82 patients with pancreatic cancer, 22 patients with chronic pancreatitis, 28 patients with benign pancreatic tumor, 88 healthy control subjects and 20 patients with colorectal cancer, gastric cancer or liver cancer each. In the blinded validation group, a panel of meaningful biomarkers selected in the training group was detected, and two combined indexes were validated based on the ROC analysis.

An independent double-blinded test was further carried out to investigate the application of two established indexes. Such double-blinded test included 10 patients with pancreatic cancer, 4 patients with chronic pancreatitis, 4 patients with benign pancreatic tumor, 90 healthy control subjects and 4 patients with colorectal cancer, gastric cancer or liver cancer each.

Independent double-blinded test was performed by an independent group. The investigators conducting the molecular analysis on the plasma samples or analysis of disease status were blinded to the patients' information and clinical diagnosis.

### Laboratory methods

MicroRNAs were purified from plasma samples using a miRNeasy Serum/Plasma Kit (QIAGEN, Germany). Cel-miR-39 was spiked into each sample as a control. The miScript SYBR Green PCR Kit (QIAGEN, Germany) was used to conduct real-time PCR on all samples to detect the expression of miRNAs with LightCycler 480 (Roche, Germany). A standard curve of cel-miR-39 was made to calculate the copy numbers of each miRNA. The human MIC-1 ELISA kit (R&D Systems, UK) and the human CA19-9 detection Kit (Roche Diagnostics GmbH, Germany) were used to detect the plasma MIC-1 and CA19-9 respectively according to the standard operating procedures, using Cobas E601 automatic electrochemical luminescence immunity analyzer (Roche, Germany). All experiments were performed in triplicates. The concordance within 10% was required.

### Statistical analyses

In the training group, the expression of six candidate miRNAs, MIC-1 and CA19-9 were detected while only those significantly elevated (miR-21, miR-25, MIC-1 and CA19-9) were evaluated in the blinded validation group and double-blinded test. Copy numbers of miRNAs were ln-transformed owing to the huge variation from 10^3^ to 10^7^, and the concentrations of MIC-1 and CA19-9 were ln-transformed as well.

Analysis of variance (ANOVA) or chi-square was initially conducted to determine the difference between the clinical characteristics of PC patients and healthy control subjects or other control groups. In the training group, plasma expression levels of miRNAs, MIC-1, and CA19-9, differences between PC group and control groups (healthy control, benign pancreatic disease or other GI cancers) were analyzed by T test. Univariate logistic regression was used to evaluate candidate biomarkers to diagnose PC patients. Multivariate logistic regression models were built to quantify the risk of PC adjusting for possible confounders and baseline characteristics. The establishment of combined indexes is shown in supplementary methods.

With index1 and index2, Receiver Operating Characteristic (ROC) analysis with 95% confidence interval (CI), sensitivity, specificity, accuracy, positive predictive value (PPV), negative predictive value (NPV), positive likelihood ratio (+LR) and negative likelihood ratio (-LR) were used to assess the performance. Area under ROC curve (AUC) was also utilized to compare the combined sensitivity and specificity among the candidate biomarkers and diagnostic indexes. The difference between the account of AUC of the indexes and CA19-9 was calculated by the method described by Hanley and McNeil. The chi-square was utilized to assess the difference of sensitivity, specificity and accuracy between the indexes and CA19-9. In the double-blinded test, sensitivity, specificity and accuracy were used to assess the predictive performance compared with CA19-9 alone.

The Pearson or Spearman correlation coefficient was used to analyze the association between candidate biomarkers and qualitative or quantitative clinical characteristics in 113 PC patients. Kaplan-Meier survival analysis was conducted to analyze the prognostic value of candidate biomarkers and two combined indexes. Two-sided tests and a significance level of 0.05 were used with IBM SPSS statistics 22.0 in this study.

## SUPPLEMENTAL DATA




