# Supplementary material for: New combined microRNA and protein plasmatic biomarker panel for pancreatic cancer
Source: Oncotarget. 2016 Oct 3;7(48):80033–45. doi: 10.18632/oncotarget.12406 (PMC5346769; doi:10.18632/oncotarget.12406)
Supplement: Supplementary file 1 [file oncotarget-07-80033-s001.pdf]

New combined microRNA and protein plasmatic biomarker panel for pancreatic cancer

SUPPLEMENTAL DATA

PATIENTS

Totally 1078 plasma samples were collected by convenience and with the written informed consents from the participants for this study. All samples but part of those from patients with chronic pancreatitis were collected at Cancer Hospital, Chinese Academy of Medical Sciences (CAMS) from March 2012 to May 2015. Several blood samples from patients with chronic pancreatitis were collected at Peking Chaoyang Hospital from March 2012 to December 2014.

The inclusion criteria were adopted as follows: All blood samples were collected from the patients who were newly diagnosed and pretreated. Patients with acute infectious biliary pancreatic disease were excluded. Lipemic and hemolyzed blood samples were rejected. Finally, 438 plasma samples were excluded and 640 samples were used in this study.

The final diagnosis of pancreatic cancer was based on the histological evaluation of surgically resected tissue specimens, cytological evaluation of intraoperative fine needle biopsy (FNA) or endoscopic ultrasound guided fine needle biopsy (EUS-FNA). The final diagnosis of CRC, GC and HCC was all based on the histological evaluation of surgically resected tissue specimens. All the healthy controls were determined to be negative in the CA19-9 detection and computed tomography, magnetic resonance imaging and/or endoscopic ultrasonography.

The tumor staging was defined according to the 7th Cancer Staging References issued by American Joint Committee on Cancer (AJCC). For the patients who underwent surgery, definitive tumor stage was established on the basis of operative findings. For those unsuitable for surgical management, the tumor stages were determined by means of computed tomography, magnetic resonance imaging and/or endoscopic ultrasonography.

METHODS

Samples collection and RNA isolation details

The blood was collected in EDTA tubes (BD Biosciences) and processed to isolate plasma. The total RNA was isolated with the miRNeasy Serum/Plasma Kit (QIAGEN, Germany) according to the manufacturer's protocol. 3.5μl miRNeasy Serum/Plasma Spike-In Control (cel-miR-39, QIAGEN, $1.6*10^8$ copies/μl) was spiked into each sample (200μl) as a control after initial plasma denaturation for RNA isolation. Each reverse-transcription reaction(20μl) was performed with 1.5μl total RNA with the miScript Reverse Transcription Kit II(QIAGEN, Germany).

Specific miRNA primers

The expression of miRNAs was detected with the miScript SYBR Green PCR Kit(QIAGEN, Germany) and specific miRNA primers (QIAGEN, Germany). The cat. No. of miR-20A specific miRNA primer assay is MS00003199, miR-21 MS00009079, miR-25 MS00003227, miR-155 MS00031486, miR-196A MS00031563, miR-210 MS00003801 and cel-miR-39 MS00019789.

Detection of MIC-1 and CA19-9

The MIC-1 ELISA kits (R&D Systems, UK) were used according to the manufacturer's instructions. The CA19-9 detection Kit (Roche Diagnostics GmbH, Germany) and Cobas E601 automatic electrochemical luminescence immunity analyzer (Roche, Germany) were used to detect the plasma CA19-9 according to the standard operating procedures.

Index establishment based on multivariate logistic regression model

Based on 5 candidate specific biomarkers selected in the training group, multivariate logistic regression models were built to quantify the risk of pancreatic cancer. Two indices were built finally. In the logistic regression models, we assumed a linear association between the ln-transformed concentration of biomarkers and disease status on logit scale.

Our two indices were linear predictors established based on multivariate logistic regression model, each index consisted of three biomarkers: miRNA (miR-21 or miR-25), MIC-1 and CA19-9. Index-n=c+β_1 x miR-X+β_2 x MIC-1+β_3x CA19-9. Where c is a constant and β [1-3] are

estimated coefficients from multivariate logistic regression model. miR-X (miR-21 or miR-25), MIC-1 and CA19-9 are log(e)-transformed concentration values for each biomarker. The prediction possibility was used to evaluate patients' disease status, *P*(Index-n)=Exp(Index-n)/(1+ Exp(Index-n)), the cutoff value was set at 0.5.

Baseline characteristics such as age, gender, BMI were not included because of no significant difference between patients with pancreatic cancer and control group. MiR-20A was removed because it made no difference in the two indices.

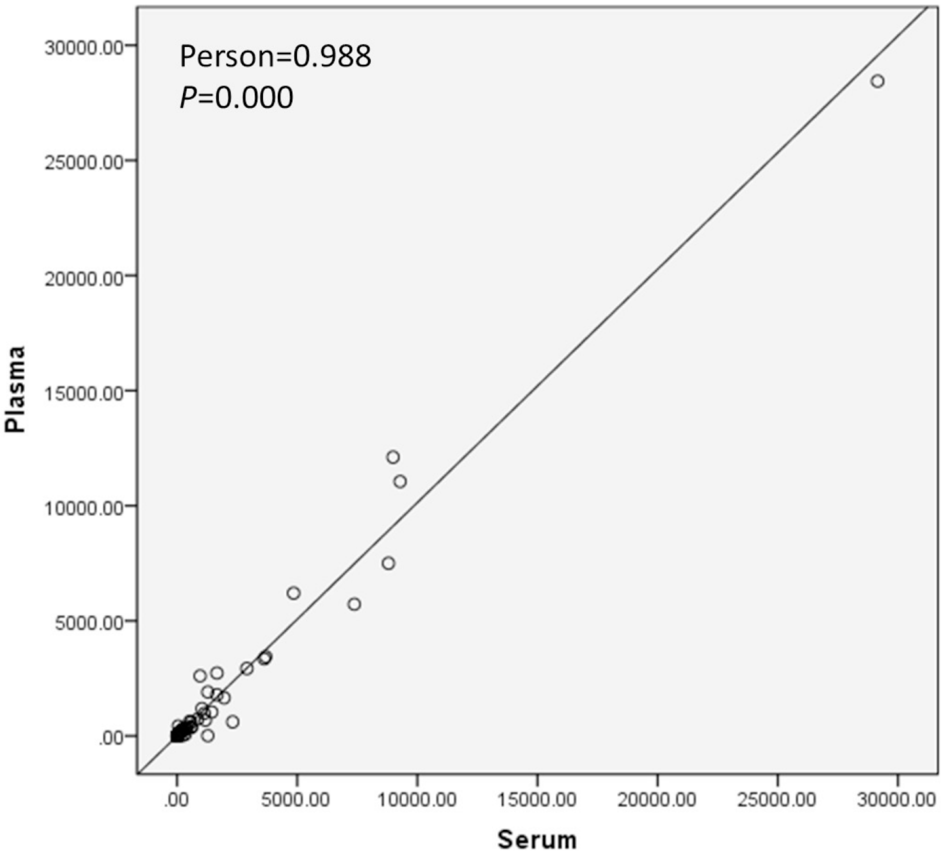

Supplementary Figure S1: Relationship of plasma CA19-9 and serum CA19-9. Person relationship analysis results showed that there's a strong correlation between our plasma CA19-9 expression and serum CA19-9 expression in clinic detection. No significant difference between plasma CA19-9 expression and serum CA19-9 expression was observed by paired T-test.

Supplementary Table S1: Basic characteristics of patients with benign pancreatic disease and other GI cancer

See Supplementary File S1.

Supplementary Table S2: Univariate logistic regression and receiver operating characteristics (ROC) analysis of each tissue specific biomarker in Training and Blinded validation Group

**Training group**	**PC VS HC**			**PC VS BPD(CP)**			**PC VS other GI cancer**		
	**OR(95%CI)**	**P value**	**AUC(95%CI)**	**OR(95%CI)**	**P value**	**AUC(95%CI)**	**OR(95%CI)**	**P value**	**AUC(95%CI)**
miR-20A	2.092(1.566-2.795)	<0.001	0.764(0.691-0.836)	1.899(1.206-2.992)	0.006	0.715(0.611-0.819)	1.876(1.348-2.611)	<0.001	0.705(0.616-0.793)
miR-21	13.392(5.902-30.386)	<0.001	0.903(0.855-0.952)	7.281(2.859-18.542)	<0.001	0.852(0.767-0.937)	4.020(2.220-7.279)	<0.001	0.780(0.700-0.860)
miR-25	5.228(2.933-9.317)	<0.001	0.790(0.719-0.861)	4.986(2.090-11.892)	<0.001	0.797(0.710-0.885)	3.657(2.079-6.430)	<0.001	0.752(0.669-0.835)
miR-196A	1.662(1.312-2.106)	<0.001	0.695(0.613-0.777)	1.117(0.787-1.584)	0.536	0.539(0.423-0.656)	0.728(0.547-0.969)	0.030	0.416(0.319-0.513)
MIC-1	20.527(8.026-52.499)	<0.001	0.917(0.871-0.963)	3.907(1.819-8.393)	<0.001	0.778(0.681-0.874)	6.344(3.293-12.224)	<0.001	0.846(0.782-0.910)
CA19-9	4.744(2.773-8.117)	<0.001	0.914(0.861-0.968)	2.349(1.572-3.512)	<0.001	0.874(0.806-0.941)	2.928(2.024-4.237)	<0.001	0.876(0.813-0.939)
**Blinded validation group**	PC VS HC			PC VS BPD(CP+BPT)			PC VS GI other cancer		
	OR(95%CI)	P value	AUC(95%CI)	OR(95%CI)	P value	AUC(95%CI)	OR(95%CI)	P value	AUC(95%CI)
miR-21	7.304(3.570-14.941)	<0.001	0.809(0.743-0.875)	3.801(2.076-6.961)	<0.001	0.743(0.660-0.827)	1.831(1.187-2.825)	0.006	0.635(0.543-0.726)
miR-25	2.349(1.515-3.643)	<0.001	0.655(0.570-0.740)	2.162(1.422-3.285)	<0.001	0.679(0.587-0.771)	1.776(1.173-2.689)	0.007	0.631(0.540-0.722)
MIC-1	14.709(6.696-32.574)	<0.001	0.896(0.849-0.943)	3.342(1.894-5.897)	<0.001	0.731(0.647-0.815)	3.729(2.186-6.363)	<0.001	0.761(0.683-0.838)
CA19-9	4.203(2.612-6.764)	<0.001	0.898(0.848-0.948)	2.086(1.544-2.819)	<0.001	0.821(0.749-0.894)	2.304(1.666-3.186)	<0.001	0.829(0.761-0.8897)

Abbreviation: OR, odds ratio; CI, confidence interval; AUC, area under the curve; PC, pancreatic cancer; HC, healthy controls; CP, chronic pancreatitis; BPT, benign pancreatic tumor; BPD, benign pancreatic disease; GI, gastrointestinal.

Supplementary Table S3: Multivariable analysis of candidate biomarkers to predict pancreatic cancer in the training group

	Multivariable analysis (Adjusted OR:95%CI)			
	PC VS CP&HC		PC VS other GI cancer	
	Index1	Index2	Index1*	Index2*
miR-20A	/	/	/	/
miR-21	6.020(2.343-15.469)	/	2.879(1.389-5.965)	/
miR-25	/	5.884(2.154-16.072)	/	3.355(1.639-6.868)
MIC-1	7.177(2.541-20.269)	8.483(3.020-23.831)	5.043(2.187-11.628)	5.356(2.266-12.656)
CA19-9	2.774(1.662-4.629)	3.082(1.876-5.061)	2.463(1.656-3.663)	2.508(1.674-3.757)

Abbreviation: OR, odds ratio. Adjusted OR: Adjusted for the combination of other biomarker in the index. Index1* and Index2* were abandoned in further analysis.

Supplementary Table S4: New indexes could diagnose both PC patients with CA19-9 positive and negative

PC Cases			CA19-9	
			+ (>37 KU/L)	– (<37 KU/L)
Training group (N=76)			N=62	N=14
	Index1	Positive(N=68)	60	8
		False negative(N=8)	2	6
	Index2	Positive(N=68)	60	8
		False negative(N=8)	2	6
Blinded validation group (N=82)			N=59	N=23
	Index1	Positive(N=72)	55	17
		False negative(N=10)	4	6
	Index2	Positive(N=69)	53	16
		False negative(N=13)	6	7

Supplementary Table S5: Correlation analysis between the expression of candidate biomarkers and two indices with clinical characteristics in patients with pancreatic cancer

Factors	miR-20A	miR-21	miR-25	miR-155	miR-196A	miR-210	MIC-1	CA19-9	Index1	Index2
Gender [a]	0.082	0.872	0.788	0.393	0.374	0.908	0.907	0.190	0.403	0.347
Metastasis[a]	0.354	0.860	0.765	0.884	0.757	0.281	0.827	0.330	0.563	0.500
Resectable[a]	0.802	0.412	0.828	0.002	0.123	0.063	0.965	0.207	0.236	0.332
Hypertension[a]	0.950	0.368	0.298	0.716	0.504	0.821	0.054	0.316	0.042	0.027
Diabetes[a]	0.977	0.291	0.185	0.303	0.595	0.333	0.518	0.075	0.053	0.030
Smoking[a]	0.318	0.397	0.256	0.615	0.432	0.739	0.921	0.254	0.772	0.855
Alcohol drinking[a]	0.754	0.899	0.294	0.236	0.484	0.783	0.733	0.336	0.467	0.733
Family cancer history[a]	0.984	0.393	0.952	0.808	0.372	0.895	0.158	0.680	0.394	0.606
Stage(I, II VS III) [a]	0.703	0.294	0.680	0.003	0.253	0.108	0.992	0.363	0.321	0.450
Stage(I, II VSIV)[a]	0.882	0.993	0.724	0.042	0.024	0.053	0.989	0.212	0.427	0.485
BMI[b]	0.317	0.274	0.812	0.095	0.782	0.342	0.745	0.421	0.850	0.545
Age[b]	0.996	0.930	0.323	0.954	0.199	0.694	0.075	0.226	0.084	0.025

aANOVA; b Pearson correlation coefficient. For miR-20A, miR-155, miR-196A and miR-210, n=113 (The expression of these four miRNAs were detected only in 113 PC samples, which had followed up information.); for miR-21, miR-25, MIC-1, CA19-9, Index1 and Index2, n=168. P values were presented in this table.

Supplementary Table S6: The expression of all biomarkers and indexes was not related with PC prognosis

Factor	Median Survival Time(month,95%CI) (N=97)		*P* value (log-rank test)
	Low expression(N=49)	High expression(N=48)	
miR-20A	7.30(6.82-7.78)	8.90(6.98-10.82)	0.534
miR-21	7.30(5.94-8.66)	8.30(5.69-10.91)	0.768
miR-25	7.30(6.04-8.56)	8.30(4.73-11.87)	0.759
miR-155	7.80(4.97-10.63)	7.50(6.01-8.99)	0.283
miR-196A	7.10(4.50-9.70)	7.70(6.59-8.82)	0.794
miR-210	7.10(6.42-7.78)	8.90(6.98-10.82)	0.135
MIC-1	7.70(4.87-10.53)	7.50(5.03-9.97)	0.842
CA19-9	7.80(5.65-9.95)	7.3(6.026-8.574)	0.130
Index1	7.80(5.96-9.64)	7.30(5.01-9.59)	0.874
Index2	8.80(6.54-11.06)	7.30(5.93-8.67)	0.221

Kaplan-Meir analysis was used to evaluate the prognostic value of candidate biomarkers.
