# Supplementary material for: New combined microRNA and protein plasmatic biomarker panel for pancreatic cancer
Source: Oncotarget. 2016 Oct 3;7(48):80033–45. doi: 10.18632/oncotarget.12406 (PMC5346769; doi:10.18632/oncotarget.12406)
Supplement: Supplementary file 2 [file oncotarget-07-80033-s002.doc]

**Supplementary table 1** Basic characteristics of patients with benign pancreatic disease and other GI cancer.

|  | No. (%) of Patients and Healthy Participants of each group | | | | | |
| --- | --- | --- | --- | --- | --- | --- |
|  | BPD | | Other GI cancer | | | |
|  | CP(n=48) | BPT(n=32) | CRC(n=44) | GC (n=44) | | HCC(n=44) |
| **Training group(n=240)** | 22(9.17) | 0(0.00) | 20(8.33) | 20(8.33) | | 20(8.33) |
| Age, median (range),y | 52.00(21.00-80.00) |  | 62.50(49.00-75.00) | 62.50(41.00-75.00) | | 51.00(34.00-76.00) |
| Gender |  |  |  |  | |  |
| Male | 10(45.45) |  | 16(80.00) | 16(80.00) | | 12(60.00) |
| Female | 12(54.55) |  | 4(20.00) | 4(20.00) | | 8(40.00) |
| Resection of tumors |  |  |  |  | |  |
| Yes | / |  | 20(100.00) | 20(100.00) | | 17(85.00) |
| No | / |  | 0 | 0 | | 3(15.00) |
| Metastasis |  |  |  |  | |  |
| Yes | / | 15(75.00) | 14(70.00) | 5(25.00) | |  |
| No | / |  | 5 (25.00) | 6(30.00) | | 15(75.00) |
| Cancer stage(AJCC) |  |  |  |  | |  |
| Ⅰ | / |  | 2(10.00) | 4(20.00) | | 7(35.00) |
| Ⅱ | / |  | 3(15.00) | 2(10.00) | | 5(25.00) |
| Ⅲ | / |  | 12(60.00) | 13(65.00) | | 4(20.00) |
| Ⅳ | / |  | 3(15.00) | 1(5.00) | | 4(20.00) |
| Hypertension |  |  |  |  | |  |
| Yes | 6(27.27) | 7(35.00) | 6(30.00) | 2(10.00) | |  |
| No | 16(72.73) |  | 13(65.00) | 14(70.00) | | 18(90.00) |
| Diabetes |  |  |  |  | |  |
| Yes | 6(27.27) |  | 4(20.00) | 4(20.00) | | 3(15.00) |
| No | 16(72.73) |  | 16(80.00) | 16(80.00) | | 17(85.00) |
| Cancer heritage |  |  |  |  | |  |
| Yes | 0(0.00) |  | 2(10.00) | 0(0.00) | | 8(40.00) |
| No | 22(100.00) |  | 18(90.00) | 20(100.00) | | 12(60.00) |
| Smoking |  |  |  |  | |  |
| Yes | 8(36.36) |  | 9(45.00) | 11(55.00) | | 3(15.00) |
| No | 14(63.64) |  | 11(55.00) | 9(45.00) | | 17(85..00) |
| Alcohol drinking |  |  |  |  | |  |
| Yes | 5(22.73) |  | 6(30.00） | 6(30.00） | | 3(15.00) |
| No | 17(77.27) |  | 14(70.00) | 14(70.00) | | 17(85..00) |
| BMI, median (range) | 23.66(19.49-28.73) |  | 25.94(21.22-29.76) | 23.14(19.36-29.67) | | 23.93(20.62-31.20) |
| Plasma CA19-9, median(range), KU/L | 22.03(2.96-53.9) |  | 15.38(4.51-30.72)  13.01(4.67-315.0) | 24.72(4.62-153.00) | |  |
| Serum CEA, median(range), μg/L | 1.00(1.00-4.86) |  | 3.87(0.99-32.36) | 1.84(0.22-251.4) | | 2.66(0.90-22.41) |
| **Validation group (n = 280)** | 22(7.86) | 28(10.00) | 20(7.14) | 20(7.14) | | 20(7.14) |
| Age, median (range) | 48.50(29.00-70.00)  49.00(16.00-71.00) | 63.00(51.00-83.00) | 50.50(30.00-78.00) | 54.50(33.00-83.00) | |  |
| Gender |  |  |  |  | |  |
| Male | 17(77.27) | 6(21.43) | 9(45.00) | 10(50.00) | | 15(75.00) |
| Female | 5(22.73) | 22(78.57) | 11(55.00) | 10(50.00) | | 5(25.00) |
| Resection of tumors |  |  |  |  | |  |
| Yes | / | 28(100.00) | 20(10.00) | 20(10.00) | | 20(10.00) |
| No | / | 0(0.00) | 0(0.00) | 0(0.00) | | 0(0.00) |
| Metastasis |  |  |  |  | |  |
| Yes | / | 0(0.00) | 8(40.00) | 12(60.00) | | 5(25.00) |
| No | / | 28(100.00) | 12(60.00) | 8(40.00) | | 15(75.00) |
| Cancer stage |  |  |  |  | |  |
| Ⅰ | / | / | 2(10.00) | 4(20.00) | | 8(40.00) |
| Ⅱ | / | / | 10(50.00) | 4(20.00) | | 7(35.00) |
| Ⅲ | / | / | 7(35.00) | 11(55.00) | | 4(20.00) |
| Ⅳ | / | / | 1(5.00) | 1(5.00) | | 1(5.00) |
| Hypertension |  |  |  |  | |  |
| Yes | 6(27.27) | 7(25.00) | 9(45.00) | 4(20.00) | | 3(15.00) |
| No | 16(72.73) | 21(75.00) | 11(55.00) | 16(80.00)  17(85.00) | |  |
| Diabetes |  |  |  |  | |  |
| Yes | 4(18.18) | 2(7.14) | 4(20.00) | 4(20.00) | | 2(10.00) |
| No | 18(81.82) | 26(92.86) | 16(80.00) | 16(80.00) | | 18(90.00) |
| Cancer heritage |  |  |  |  | |  |
| Yes | 2(9.09) | 2(7.14) | 6(30.00) | 6(30.00) | | 4(20.00) |
| No | 20(90.91) | 26(92.86) | 14(70.00) | 14(70.00) | | 16(80.00) |
| Smoking |  |  |  |  | |  |
| Yes | 5(22.73) | 2(7.14) | 6(30.00) | 4(20.00) | | 6(30.00) |
| No | 17(77.27) | 26(92.86) | 14(70.00) | 16(80.00) | | 14(70.00) |
| Alcohol drinking |  |  |  |  | |  |
| Yes | 3(13.64) | 2(7.14) | 6(30.00) | 6(30.00) | | 4(20.00) |
| No | 19(86.36) | 26(92.86) | 14(70.00) | 14(70.00) | | 16(80.00) |
| BMI, median (range)  26.09(22.49-30.56) | 24.51(19.49-30.83) | 25.01(20.04-29.76) | 23.66(17.63-30.02) | 24.19(16.82-32.01) | |  |
| Plasma CA19-9, median(range), KU/L | 24.85(2.70-2012.00) | 8.50(3.54-20.38) | 11.87(0.60-160.1) | 13.12(1.50-1446.00) | | 16.31(1.97-86.65) |
| Serum CEA, median(range) μg/L  1.48(0.98-17.38) | 1.30(0.36-3.42) | 3.42(1.18-38.85) | 1.48(0.56-29.64) | 1.84(0.80-7.58) | |  |
| **Screening test** | 4(3.33) | 4(3.33) | 4(3.33) | 4(3.33) | | 4(3.33) |
| Age, median (range) | 54.00(48.00-61.00) | 45.00(39.00-48.00) | 59.00(49.00-70.00) | 53.00(45.00-70.00) | | 56.50(40.00-59.00) |
| Gender |  |  |  |  | |  |
| Male | 2(50.00) | 2(50.00) | 2(50.00) | 3(75.00) | | 3(75.00) |
| Female | 2(50.00) | 2(50.00) | 2(50.00) | 1(25.00) | | 1(25.00) |
| Resection of tumors |  |  |  |  | |  |
| Yes | / | 4(100.00) | 4(100.00) | 4(100.00) | | 4(100.00) |
| No | / | 0(0.00) | 0(0.00) | 0(0.00) | | 0(0.00) |
| Metastasis |  |  |  |  | |  |
| Yes | / | 4(100.00)  1(25.00) | 2(50.00) | 1(25.00) | |  |
| No | / | 0(0.00) | 3(75.00) | 2(50.00) | | 3(75.00) |
| Cancer stage |  |  |  |  | |  |
| Ⅰ | / | / | 1(25.00) | 1(25.00) | | 1(25.00) |
| Ⅱ | / | / | 2(50.00) | 1(25.00) | | 2(50.00) |
| Ⅲ | / | / | 1(25.00) | 1(25.00) | | 1(25.00) |
| Ⅳ | / | / | 0(0.00) | 1(25.00) | | 0(0.00) |
| Hypertension |  |  |  |  | |  |
| Yes | 1(25.00)  0(0.00) | 0(0.00) | 0(0.00) | 1(25.00) | |  |
| No | 3(75.00) | 4(100.00) | 4(100.00) | 4(100.00) | | 3(75.00) |
| Diabetes |  |  |  |  | |  |
| Yes | 1(25.00) | 0(0.00) | 0(0.00) | 0(0.00) | | 1(25.00) |
| No | 3(75.00) | 4(100.00) | 4(100.00) | 4(100.00) | | 3(75.00) |
| Cancer heritage |  |  |  |  | |  |
| Yes | 0(0.00) | 0(0.00) | 0(0.00) | 0(0.00) | | 1(25.00) |
| No | 4(100.00) | 4(100.00) | 4(100.00) | 4(100.00) | | 3(75.00) |
| Smoking |  |  |  |  | |  |
| Yes | 0(0.00) | 1(25.00) | 0(0.00) | 0(0.00) | | 2(50.00) |
| No | 4(100.00) | 3(75.00) | 4(100.00) | 4(100.00) | | 2(50.00) |
| Alcohol drinking |  |  |  |  | |  |
| Yes | 0(0.00) | 1(25.00) | 1(25.00) | 1(25.00) | | 3(75.00) |
| No | 4(100.00) | 3(75.00) | 3(75.00) | 3(75.00) | | 1(25.00) |
| BMI, median (range) | 22.45(19.89-25.56) | 23.68(19.31-25.71) | 25.05(21.35-27.13) | 25.64(24.74-26.90) | | 25.11(18.69-25.43) |
| 1.37(1.04-4.53)5.29(0.32-23.58)2.06(0.73-4.27)3.02(2.07-4.85)Plasma CA19-9, median(range), KU/L | 47.92(10.25-129.40) | 6.83(2.53-25.74) | 26.80(7.66-29.13) | 19.25(8.44-39.45) | | 47.41(37.70-51.33) |
| 1.67(1.00-2.15)  Serum CEA, median(range) μg/L | | | | |  | |

Abbreviation:PC, pancreatic cancer; HC, healthy controls; CP, chronic pancreatitis; BPT, benign pancreatic tumor; CRC, colorectal cancer; GC, gastric cancer; HCC, hepatocellular carcinoma.
